# Potential of R Wave in aVL Lead in Cardiovascular Risk Assessment

**DOI:** 10.3390/biomedicines14040905

**Published:** 2026-04-16

**Authors:** Juraj Jug, Martina Lovrić Benčić, Tomislav Bulum, Ingrid Prkačin

**Affiliations:** 1 Family Medicine Department, Health Center Zagreb-West, 10000 Zagreb, Croatia; 2 School of Medicine, University of Zagreb, 10000 Zagreb, Croatia; 3 Department for Cardiovascular Diseases, University Hospital Center Zagreb, 10000 Zagreb, Croatia; 4 Vuk Vrhovac University Clinic for Diabetes, Endocrinology and Metabolic Diseases, Merkur University Hospital, 10000 Zagreb, Croatia; 5 Department for Internal Medicine, Medikol Polyclinic, 10000 Zagreb, Croatia; ingrid.prkacin@gmail.com

**Keywords:** arterial hypertension, arterial stiffness, cardiovascular risk, electrocardiography, risk factors

## Abstract

**Background:** R wave amplitude in the aVL ECG lead (R_aVL_) has been identified as a marker of cardiovascular (CV) risk, hypertension-mediated target organ damage (HMOD), and mortality in patients with arterial hypertension (AH), where R_aVL_ > 1.1 mV suggests left ventricular hypertrophy. However, the exact threshold for identifying high-risk patients has yet to be determined. Therefore, we compared R_aVL_ values among hypertensive patients with and without hypertensive urgencies (HUs) and healthy subjects, aiming to identify the predictors of elevated R_aVL_ and to compare its prognostic value with the SCORE 2 model. **Methods:** This cross-sectional study included 339 participants divided into three groups according to ambulatory blood pressure monitoring results: 100 patients with AH and HU from the emergency department, 134 patients with AH without HU, and 105 healthy subjects recruited from four family medicine practices. Basic laboratory parameters were determined, SCORE 2 risk was calculated, PWV was measured using oscillometry, and a standard 12-lead ECG was recorded in all participants. **Results:** Participants with AH and HU had the highest R_aVL_ values compared to those with AH without HU and healthy subjects (averages of 0.76 ± 0.24 mV, 0.49 ± 0.27, 0.22 ± 0.25, respectively; *p* < 0.001). Significantly higher R_aVL_ values were observed in males compared to females (0.56 ± 0.31 vs. 0.41 ± 0.34 mV; *p* < 0.001) and in non-dippers compared to dippers (0.56 ± 0.34 mV vs. 0.41 ± 0.31 in dippers; *p* < 0.001). Age, mean arterial pressure, PWV, and SCORE 2 were shown as independent predictors of R_aVL_. Compared with SCORE 2, individuals with R_aVL_ > 0.40 mV had high CV risk (sensitivity of 58.16%, specificity of 73.68%; *p* < 0.001). **Conclusions:** In this study, R_aVL_ demonstrated good prognostic value for CV risk stratification. However, larger studies are needed to determine a precise high-risk threshold to improve CV risk estimation and HMOD detection in patients with marginal SCORE 2 CV risk.

## 1. Introduction

The amplitude of the R wave in lead aVL of the ECG (R_aVL_) is an old measure of left ventricular hypertrophy (LVH) described and proposed in 1949 by Maurice Sokolow and Thomas P. Lyon [1]. In fact, R_aVL_ reflects the sum of the largest part of the vectors generated by electrical activation of the left ventricle and can be used as a marker of cardiac remodeling [2]. However, obese and elderly individuals, due to a heart position change towards the horizontal, may have higher R_aVL_ amplitudes [1]. According to current ESH guidelines, with a specificity of 97% but low sensitivity (40–50%), a R_aVL_ value > 1.1 mV is characteristic of LVH and hypertension-mediated organ damage (HMOD) in patients with arterial hypertension (AH) [3,4].

Only a few important studies on this topic have been conducted. After a ten-year follow-up of 596 patients with AH, Courand et al. concluded that patients with R_aVL_ > 0.6 mV have a high cardiovascular (CV) risk [5,6]. A year later, the same researchers demonstrated, using cardiac magnetic resonance imaging, that a R_aVL_ value > 1.0 mV is indicative of LVH, while for R_aVL_ values between 0.5 and 1.0 mV, they suggest using other indices to assess the presence of LVH [7]. Grandjean et al. went a step further in 2019, showing that a R_aVL_ > 0.7 mV with an elevated serum NTproBNP value > 150 pg/mL (17.74 pmol/L) had a prognostic value for higher CV mortality over 10 years, regardless of LVH [8]. However, no studies have compared the predictive value of R_aVL_ for increased CV risk with that of pulse wave velocity (PWV) or the SCORE 2 model, and its thresholds for CV risk determination have not yet been determined in the guidelines.

Of the 35 existing electrocardiographic criteria for LVH, the Sokolow–Lyon and Cornell voltage criteria are the most commonly used today, and their association with CV risk in some studies is weak [5,9]. However, in other studies, these criteria are associated with a chance of sudden cardiac death, while with higher values of the Sokolow–Lyon criteria, risk of stroke is higher in women [10,11]. In the results of the SARA study, the best detection of LVH was shown to be a combination of the Sokolow–Lyon voltage criterion (index) or the product with the Cornell product [12]. Antihypertensive therapy, i.e., good control of AH, can lead to regression of LVH, a decrease in PWV value, and a reduction in CV risk [13,14,15,16,17,18].

Around 40% of the world’s population has AH; half of them are unaware that they have it, and only a third achieve good blood pressure (BP) control (<140/90 mmHg) [19]. Poor therapeutic adherence, clinical inertia, excessive salt intake, and stress can lead to marked BP elevations and hypertensive crisis (defined as BP > 180 and/or 110 mmHg) [20]. Although all patients with these BP values are classified as having high CV risk, not all have HMOD, and about half are asymptomatic (hypertensive urgency [HU]). These patients should be evaluated promptly and treated accordingly, but current guidelines remain inconclusive [21].

PWV is a measure of arterial stiffness (level of distensibility) and is associated with the extent of atherosclerosis and mean arterial pressure (MAP). Long-lasting unregulated AH (especially with common HUs), hyperglycemia, hypercholesterolemia, obesity, and smoking cause arterial wall inflammation and accelerate structural changes (stiffening) of all arteries through collagen accumulation and elastin/fibrin degradation, leading to fibrosis and, consequently, increased PWV [22,23]. Elevated PWV alters perfusion in the coronary circulation and other parenchymal organs, resulting in impaired autoregulation and HMOD. Thus, chronic exposure to high arterial stiffness and MAP can cause cardiac fibrosis, concentric LVH (HMOD), and increased R_aVL_. Consequently, all of these factors are associated with high CV risk, and may be enable more accurate risk assessment than the SCORE 2 model alone [24].

The SCORE 2 model incorporates only age, smoking status, non-HDL cholesterol, and systolic BP values in CV risk estimation [25]. Therefore, BP is the only parameter influencing both SCORE 2 and PWV. To address this limitation, the ESH guidelines recommend carotid–femoral PWV measurement in all patients with AH for early HMOD detection, with 10 m/s defined as the threshold for high risk [4]. Several studies have demonstrated that CV risk assessment based on PWV is more accurate than SCORE, as PWV reflects the cumulative impact of all CV risk factors on the arterial wall over the patient’s lifetime [26,27]. Accordingly, each 1 m/s increase in PWV value above the reference value is associated with a 14% higher CV risk [28,29].

In some cases, estimating CV risk can be challenging and often requires clinical experience and multiple estimation methods. Reclassification of a patient’s CV risk (for example, by using R_aVL_), e.g., from moderate to high, may indicate a need for drug treatment of risk factors, whereas, according to the SCORE 2 model, only lifestyle changes would be encouraged [15]. The discovery and application of new knowledge to estimate CV risk in practice are key to the early prevention of CV events, the timely initiation of therapy, and the targeted referral of patients to other specialist physicians. PWV measurement is often time-consuming and has limited availability. However, ECG is routinely performed in all health centers and hospitals, making R_aVL_ a practical additional factor for CV risk assessment, especially in patients with HUs. Yet its exact high-risk threshold remains unknown. We hypothesized that R_aVL_ has a good prognostic value in CV risk estimation in patients with AH, regardless of LVH. Therefore, we compared R_aVL_ values across three groups (subjects without AH, those with regulated AH, and those with HUs), aiming to identify the predictors of elevated R_aVL_ and to compare its prognostic value for CV risk estimation with the SCORE 2 model.

## 2. Materials and Methods

This cross-sectional study was conducted from January 2019 to December 2022 in Zagreb, Croatia. It included four family medicine practices (FMPs) at the Health Center Zagreb-West (groups A and B) and the Emergency department (ER) at Clinical Hospital Merkur (group C). Participants from FMPs were randomly selected from a list of patients according to the appropriate criteria listed in Section 2.1. Patients from the ER were enlisted after the exclusion of acute HMOD (hypertensive emergency). Medical history was obtained from their available electronic medical records. On the first visit, 24 h ambulatory blood pressure monitoring (ABPM) was installed on the patient. ECG recording and PWV measurements were performed the day after, and participants were then referred to the local laboratory. The subjects were divided into three groups based on ABPM results. Power analysis required at least 100 participants per group, but certain participants had to be regrouped due to unexpected ABPM results, resulting in different numbers of patients in these groups. In the end, we included 339 Caucasian participants (175 women and 164 men): 100 with AH and HU, 134 with AH without HU, and 105 healthy subjects.

### 2.1. Groups Depending on Hypertensive Status

- Group A (healthy persons): Subjects were selected by simple random sampling from the list of insured persons in four FMPs without AH (ICD-10: I10-I15) and with average BP values lower than 130/80 mmHg as measured by ABPM.
- Group B (patients with AH without HU): Subjects from four FMPs with a diagnosis of AH (ICD-10: I10-I15) confirmed by ABPM without measured BP values higher than 180 mmHg and/or 120 mmHg in more than five individual measurements and data about HU in their medical history. AH is defined by exceeding the average of all measured BP values above 130 and/or 80 mmHg by APBM, and/or taking at least one antihypertensive drug [4,30].
- Group C (patients with HU): Formed by simple random sampling of patients from a database of patients from Clinical Hospital Merkur who sought emergency medical help due to high BP values and for whom the appropriate tests eliminated acute HMOD. BP was measured upon arrival at the institution after 5 min of quiet sitting, with a 1 min interval between measurements (three measurements total), and ABPM was set upon discharge. Only patients whose BP values were measured by a correctly performed ABPM > 180 and/or 120 mmHg in more than five individual measurements for 24 h were included.

### 2.2. Exclusion Criteria

The exclusion criteria were as follows: hypertensive emergency, CV events in personal medical history, incomplete data, age below 40 and above 70 years, BMI > 50 kg/m^2^, atrial fibrillation, diabetes mellitus, chronic kidney disease G3b, G4 and G5, pregnancy, immunosuppressive, corticosteroid and/or biological therapy, chemotherapy regime in the last 5 years, and expected life expectancy less than 6 months.

### 2.3. PWV and ABPM Measurements

ABPM was performed using a BTL Cardiopoint^®^ device (BTL Industries, Prague, Czech Republic) using an adequate cuff placed on the lower half of the subject’s upper arm, determined by the circumference of the middle part of the distal half of the upper arm. All subjects were divided into four groups according to the percentage of nightly BP decrease (extreme dipper: ≥20%; dipper: 10–20%; non-dipper: 0–10%; inverse dipper: <0%) [16]. In case of borderline BP values (e.g., more than 5 measured SBP values above 170 mmHg, etc.), a duration of ABPM shorter than 21 h, or less than 70% correct measurements, ABPM was repeated two weeks after the first measurement [30].

PWV was measured exclusively in this study using an Agedio^®^ B900 oscillometric device (IEM GmbH, Stolberg, Germany) at a room temperature of 20–25 °C with a suitable cuff. Two measurements were taken, with a 30 s break between 2 and 6 pm. At the first measurement, brachial SBP and DBP were determined; at the second, PWV, central SBP (cSBP), central DBP (cDBP), pulse pressure (PP), and augmentation index (AIx) were measured. In the event of imprecise measurement, the device reported an error and repeated the measurement within 5 min of the first measurement.

### 2.4. ECG Recording

A standard 12-channel ECG (BTL Cardiopoint^®^) was recorded under the same conditions as PWV (Section 2.3). All ECG records were interpreted by the leading investigator (JJ) and confirmed by the cardiologist (MLB). All R_aVL_ values, Sokolow–Lyon, and Cornell voltage criteria were determined digitally using BTL Cardiopoint^®^ official software 3. Sokolow–Lyon voltage criteria were calculated as the amplitude of the S wave in V1 lead plus the amplitude of the highest R wave in leads V5 and V6 (>35 mV = LVH). Cornell voltage criteria sums up the amplitude of the S wave in the V3 lead and R wave in the aVL lead (≥28 mV in men and ≥20 mV in women = LVH) [31].

### 2.5. Other Procedures

Acute organ damage in the case of HU is ruled out by standard biochemical and radiological examinations of the clinic (X-ray, CT, ultrasound with or without color Doppler) and ECG, and includes acute kidney damage, ischemic or hemorrhagic stroke, acute coronary syndrome, acute pulmonary edema, acute left heart failure, hypertensive encephalopathy, and aortic dissection. Biochemical parameters in the examinee’s serum were determined by sampling two tubes of 8–10 mL of venous blood in the emergency hospital laboratory: hematocrit value, concentration of creatinine, sodium ions, potassium ions, and glucose, while triglycerides, HDL, LDL, total cholesterol, and urate (since they cannot be done in an emergency laboratory). For other subjects, the same laboratory parameters were determined in the primary health care laboratory (including lipid status and urate concentration), and the fasting glucose value was repeated after 2 weeks. The glomerular filtration rate (eGFR) was assessed using the CKD-EPI (Chronic Kidney Disease Epidemiology Collaboration) formula [32]. Chronic antihypertensive and hypolipemic therapy was also recorded.

SCORE 2 CV risk was calculated in all patients with the model for high-risk countries (Croatia) from their age, smoking status, non-HDL cholesterol, and systolic blood pressure values [25].

### 2.6. Statistical Analysis

The normality of the data distribution was checked with the Kolmogorov–Smirnov test. Depending on the distribution, either Student’s t-test or Mann–Whitney U test was used to analyze the continuous variables. The categorical variables were analyzed with Pearson’s χ^2^ test. ANOVA was used in dependent variable analysis.

The sample size was calculated based on the expected arithmetic means and standard deviations of the PWV values. ANOVA for special effects and interactions with a significance level α = 0.05, effect size (f) = 0.25, and a total of 251 respondents with equally large groups had a test power of β = 95% to recognize differences in two dependent variables between the three observed groups with one predictor [33].

The association between variables was assessed using Pearson correlation and multivariate regression. Variables that had a value of *p* < 0.20 in the bivariate correlation analysis using the Pearson test were included as predictors in the multivariate regression analysis of the impact on PWV, R_aVL_, or SCORE 2.

To determine the optimal threshold value, a ROC (Receiver Operating Characteristic) analysis was performed with a display of the area under the ROC curve (area under the curve, AUC), specificity, sensitivity, and Youden’s index (DeLong’s analysis) for R_aVL_ in predicting CV risk compared to SCORE 2 CV risk. A *p*-value < 0.05 was considered statistically significant. Statistical analysis of the data was performed using Statistica (StatSoft Inc., Tulsa, OK, USA, version 12.0) and MedCalc (version 22.9).

## 3. Results

The average age of the subjects was 56.17 ± 7.96 years, and the average BMI was 27.82 ± 4.87 kg/m^2^. The differences between the ABPM groups are shown in Table 1. The mean BMI, Sokolow–Lyon, and Cornell indices were highest in group C, and 6 (1.77%) and 5 (1.47%) subjects, respectively, met the criteria for LVH according to these indices. On the other hand, a third of all subjects had R_aVL_ > 0.7 mV (high CV risk); eight subjects (2.36%) met the criteria for possible LVH (>1.1 mV).

The male subjects were younger than the female subjects (54.69 ± 8.09 vs. 57.55 ± 7.52 years; *p* < 0.001), had a higher BMI (28.55 ± 4.48 vs. 27.13 ± 5.13; *p* = 0.007) and had higher average BP values on ABPM (total average systolic pressure 137.18 ± 15.80 vs. 129.85 ± 17.55 mmHg; *p* < 0.001; diastolic pressure 84.41 ± 12.33 vs. 76.89 ± 10.51 mmHg; *p* < 0.001). There were no gender differences in heart rate values (mean value 73.47 ± 9.52 vs. 73.18 ± 8.60 beats/min; *p* = 0.771). In addition to physiologically higher values of serum hemoglobin and hematocrit concentrations and lower values of HDL cholesterol, men had statistically significantly higher uric acid values compared to women (376.38 ± 82.86 vs. 291.29 ± 70.72 umol/L; *p* < 0.001). R_aVL_ was significantly lower in women (0.41 ± 0.34 vs. 0.56 ± 0.31 mV; *p* < 0.001), as was the Cornell index (1.12 ± 0.57 vs. 1.40 ± 0.53 mV; *p* = 0.005).

The most commonly used antihypertensive drugs were angiotensin-converting enzyme inhibitors (ACEi) and calcium channel blockers (60% and 57% of participants, respectively). Almost 16% of participants in group B and 25% in group C did not have any antihypertensive drug prescribed, while in total, 12% of them had only one active substance prescribed (monotherapy). All other prescribed antihypertensive drugs are shown in Table 2.

**Figure 1:**
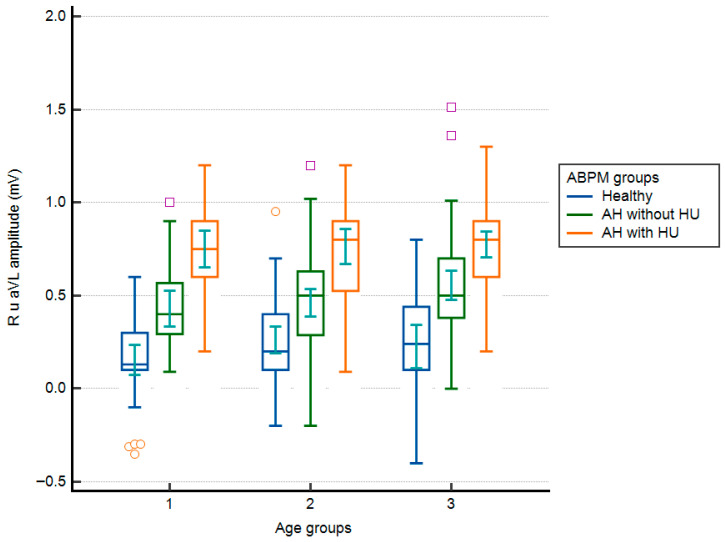
R_aVL_ values according to age groups and groups of subjects according to the results of ambulatory blood pressure measurement (box–whiskers: mean, standard error, extremes; small geometric shapes: average, standard error). AH = arterial hypertension; HU = hypertensive urgency. Age groups: 1 = 40–49 years; 2 = 50–59 years; 3 = 60–70 years.

Analysis of R_aVL_ by age group did not reveal statistically significant differences (0.41 ± 0.34 mV [40–49 years] vs. 0.47 ± 0.32 mV [50–59 years] vs. 0.56 ± 0.33 mV [60–70 years]; one-way ANOVA; F = 2.000; *p* = 0.144). However, the proportion of subjects with R_aVL_ ≥ 0.7 mV increased with age: 18 subjects (21.9%) were in the youngest age group, 38 subjects (27.9%) were in the 50–59 age group, and 48 subjects (39.7%) were in the oldest age group. Additional division of subjects by ABPM revealed statistically significant differences in R_aVL_ within the same age group. At the same time, there were no differences in R_aVL_ between the same groups of subjects, as assessed with ABPM, across age groups. In all age groups, the lowest R_aVL_ values were observed in subjects in group A (Figure 1).

### 3.1. R_aVL_ Values and Nocturnal BP Decrease

**Figure 2:**
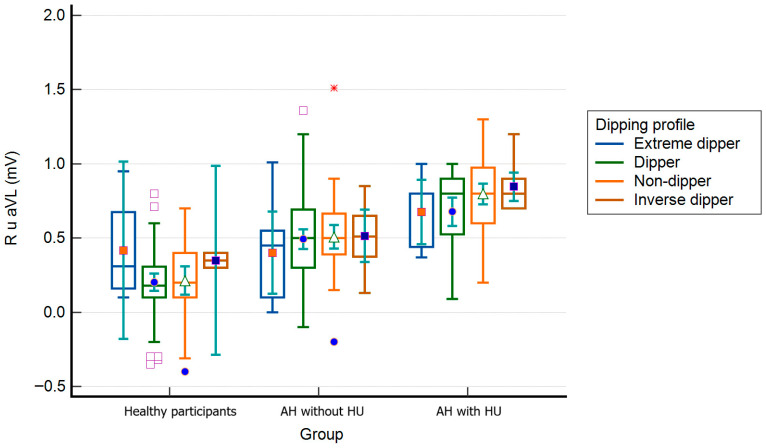
R_aVL_ values according to the ambulatory blood pressure monitoring the groups and participants’ dipping profiles (box–whiskers: mean, standard error, extremes; small geometric shapes: average, standard error). AH = arterial hypertension; HU = hypertensive urgency. * The red asterisk indicates an elevated R-wave amplitude in aVL suggesting left ventricular hypertrophy.

The lowest R_aVL_ values were found in the group of subjects with a regular dipper profile, and the highest were found in the group with an inverse dipper profile. In contrast, the differences between all subjects according to the drop in night pressure were statistically significant (0.46 ± 0.35 mV [extreme dippers] vs. 0.41 ± 0.31 mV [dippers] vs. 0.56 ± 0.34 mV [non-dippers] vs. 0.69 ± 0.26 mV [inverse dippers]; one-way ANOVA, F = 8.541; *p* < 0.001). According to the ABPM groups and the drop in night pressure in group C, the values of R_aVL_ were significantly higher in subjects with a non-dipper profile compared to those with a dipper profile (0.80 ± 0.25 vs. 0.68 ± 0.24 mV; *p* < 0.05). In group A, extreme and inverse dippers had significantly higher R_aVL_ than dippers and non-dippers (0.46 ± 0.31 vs. 0.38 ± 0.08 vs. 0.20 ± 0.25 vs. 0.21 ± 0.26 mV, *p* < 0.01). No other differences were found within APBM groups in R_aVL_ values (Figure 2).

### 3.2. Sokolow–Lyon Index

In addition to the already mentioned significant differences between the ABPM groups, the Sokolow–Lyon index of all the observed parameters was statistically significantly different only between the BMI groups, with the highest values in obese subjects and the lowest in undernourished subjects (1.75 ± 0.87 mV [undernourished] vs. 1.85 ± 0.56 mV [normal BMI] vs. 1.98 ± 0.64 mV [overweight] vs. 2.32 ± 0.49 mV [obese]; one-way ANOVA, F = 4.139; *p* = 0.007). A tendency towards higher values of the Sokolow–Lyon index was found in men (2.09 ± 0.54 mV vs. 1.94 ± 0.65 mV in women; *p* = 0.149) and in smokers (2.16 ± 0.67 mV vs. 1.98 ± 0.59 mV in non-smokers; *p* = 0.243), regardless of gender. The values of the Sokolow–Lyon index were not significantly different when compared depending on the presence of prediabetes, white coat syndrome, and the number of antihypertensive drugs used by the subject. Significant differences were found between age groups, with the highest values being found in the youngest age group (2.19 ± 0.53 mV [40–49 years] vs. 2.05 ± 0.54 mV [50–59 years] vs. 1.87 ± 0.67 mV [60–70 years]; one-way ANOVA, F = 3.095; *p* = 0.049). Factorial ANOVA by subject and age group did not reveal any significant differences (F = 0.355; *p* = 0.840).

### 3.3. Cornell Index

Unlike the Sokolow–Lyon index, the Cornell index was significantly higher in men (1.40 ± 0.53 mV vs. 1.12 ± 0.57 mV in women; *p* = 0.005) and subjects using only one antihypertensive (1.10 ± 0.50 mV vs. 1.44 ± 0.59 mV in subjects with multiple antihypertensives; *p* < 0.001). No statistically significant differences were found between the physical nutrition groups (F = 0.109; *p* = 0.955). A tendency towards higher Cornell index values was found in smokers (1.41 ± 0.47 mV vs. 1.22 ± 0.58 mV in non-smokers; *p* = 0.193) and in subjects with prediabetes (1.32 ± 0.53 mV vs. 1.19 ± 0.59 mV in subjects without prediabetes; *p* = 0.218). The Cornell index was significantly higher in the oldest age group (1.19 ± 0.52 mV [40–49 years] vs. 1.08 ± 0.47 [50–59 years] vs. 1.44 ± 0.62 [60–70 years]; one-way ANOVA, F = 3.246; *p* = 0.042), while factorial ANOVA showed no significant differences between age groups (F = 0.647; *p* = 0.630).

### 3.4. Correlations and Multivariate Analysis

According to Spearman’s correlation coefficient, the value of R_aVL_ increased less with age than with PWV (r = 0.166; *p* < 0.01), but the increase in the Cornell index was significantly higher with increasing R_aVL_, since R_aVL_ is included in the formula for its calculation (r = 0.619; *p* < 0.001). R_aVL_ increased with increasing SCORE 2 CV risk (r = 0.506; *p* < 0.001). Of the values measured by ABPM, R_aVL_ increased significantly with increasing systolic BP (r = 0.568; *p* < 0.001), diastolic BP (r = 0.492; *p* < 0.001), and MAP (r = 0.552; *p* < 0.001), but less with decreasing nocturnal blood pressure (r = −0.194; *p* < 0.001). Heart rate values increased slightly with increasing R_aVL_ (r = 0.178; *p* < 0.001). eGFR was not related to R_aVL_. In contrast to the lack of association with PWV, potassium concentration decreased with increasing R_aVL_ (r = −0.229; *p* < 0.001) as well as LDL cholesterol concentration (r = 0.115; *p* < 0.05). Among the other parameters, unlike PWV, R_aVL_ increased with the Sokolow–Lyon index (r = −0.209; *p* < 0.05).

Nine independent variables were included in the final model: the subject’s age, MAP, PWV, BMI, SCORE 2, potassium, Cornell index, gender, and eGFR. The developed model explains 49.4% of the variance in the dependent variable and is statistically significant overall. This model predicts a statistically significant increase in R_aVL_ amplitude with aging, along with increases in Cornell index, MAP, PWV, BMI, and SCORE 2 CV risk, and a decrease in potassium concentration. A tendency to decrease the R_aVL_ amplitude was found for female gender and higher eGFR (Table 3). In this model, the Sokolow–Lyon index was not an independent predictor of an increase in R_aVL_.

### 3.5. ROC Analysis

**Figure 3:**
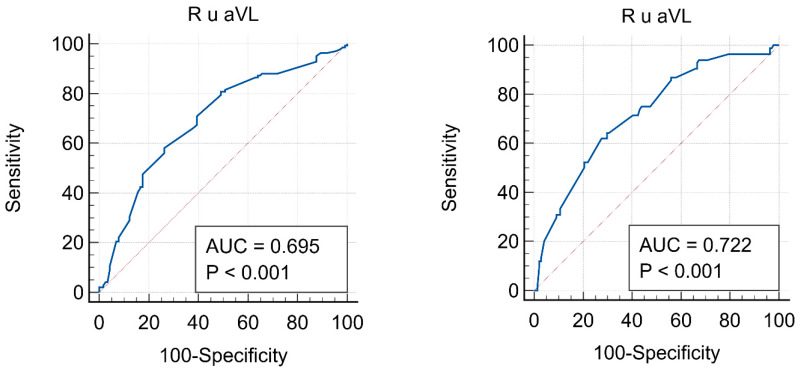
ROC curves for the R_aVL_ in the group of subjects: **left**—with high and low–moderate SCORE 2 risk; **right**—with high and very high SCORE 2 risk.

For R_aVL_, the cut-off values for high versus low–moderate CV risk were 0.4 mV (sensitivity of 58.16%, specificity of 73.68; *p* < 0.001), while the cut-off between high and very high CV risk for R_aVL_ was 0.61 mV (sensitivity of 64.29%, specificity of 70.21%; *p* < 0.001). The ROC curves for R_aVL_ according to SCORE 2 CV risk and their characteristics are shown in Figure 3.

## 4. Discussion

Subjects with AH and HU had a significantly higher R_aVL_ amplitude than patients with AH without HU. On the other hand, patients with AH without HU had significantly higher R_aVL_ amplitudes than healthy subjects. All differences were clearly expressed across all three observed age groups. Using R_aVL_ > 0.70 mV, as many as 93% of subjects in this group exceed the threshold for very high CV risk [5]. Unlike R_aVL_, the Sokolow–Lyon index was not associated with PWV values, and it decreased with increasing R_aVL_. The Cornell index increased with increasing PWV and R_aVL_, but was confirmed only as an independent predictor of increasing R_aVL_. Also, R_aVL_ was a statistically significant predictor of increased SCORE 2 CV risk. Significantly higher R_aVL_ values were observed in non-dippers and inverse dippers compared to dippers and extreme dippers. Finally, our results show that R_aVL_ can be used as an additional risk factor in CV risk stratification.

### 4.1. Cardiovascular Risk Stratification

A major problem in everyday clinical practice is the low use of tools for CV risk assessment and prevention. Although the ESH-ESC guidelines now recommend the use of the SCORE 2 model for CV risk calculation, the presence of HMOD is not included in it (same as the previous SCORE model), making this assessment quite imprecise [26]. Also, the aforementioned new guidelines expand the HMOD definition and recommend measuring cfPWV (>10 m/s) and baPWV (>18 m/s) [3,4]. The results of this study demonstrate the simplicity, precision, and feasibility of oscillometric PWV measurement within family medicine, which assesses CV risk in different individuals almost daily. According to the results of this study, if a person has R_aVL_ > 0.40 mV, HMOD should be considered (AUC = 0.695). In this scenario, individuals should be managed in accordance with guidelines for patients at high CV risk. We selected this R_avL_ cut-off using the Youden index to maximize sensitivity and specificity versus SCORE 2. However, the R_aVL_ cut-off values for CV risk groups were defined using values extrapolated from other studies [5,6]. Therefore, there are differences in the defined CV risk groups and the cut-off values obtained by ROC analysis in this study. Interestingly, the R_aVL_ cut-off values for high risk that we obtained were lower than those defined by other authors. However, due to minor differences between this study and other studies, a very high CV risk was defined as R_aVL_ > 0.7 mV. In contrast, the ROC analysis in this study identified a R_aVL_ threshold of >0.61 mV (AUC = 0.722), whereby some subjects with very high CV risk were also classified as high CV risk. Overall, based on the R_aVL_ in this study, a different proportion of subjects were classified into CV risk groups, underscoring the importance of individualized assessment of CV risk using multiple tools. Large prospective studies are needed to validate R_aVL_ cut-offs against hard CV outcomes and standardized PWV measures to define clinically actionable thresholds [28,34].

In the multivariate linear regression model, the R_aVL_ value was strongly associated with the Cornell index (β = 0.485, *p* < 0.001), as expected, since, along with the S wave in lead V3 of the ECG, it is an integral component of the formula for its calculation. This model also demonstrated the previously described dependence of the R_aVL_ value on BMI; higher MAP, higher PWV, higher SCORE 2 risk, lower potassium, and older age were independent predictors of increased R_aVL_. On the other hand, the Sokolow–Lyon index is calculated from the voltage of precordial leads and does not depend on the R_aVL_ values. Also, the Sokolow–Lyon index is more sensitive to obesity, which can explain its inverse correlation with R_aVL_ in this study. No study has examined the association between R_aVL_ and the observed variables. The results of Liao et al., who examined the prevalence of LVH in a Chinese population aged 36–45 years using the Cornell voltage product, are noteworthy. The Cornell index was shown to be associated with female sex, systolic and diastolic BP, fasting glucose, urates, and cIMT, whereas it was inversely associated with potassium concentration, as in this study [35]. Lower potassium concentration was shown to be an independent predictor of increased Cornell product, i.e., LVH, during treatment with antihypertensive therapy [36].

Precisely because of its simplicity, reproducibility, and only minor dependence on gender and BMI, in 2009, the American Heart Association (AHA) classified R_aVL_ among 35 the ECG criteria for assessing the presence of LVH, which generally has very low sensitivity and high specificity. Of the commonly used ECG criteria for LVH, the sensitivity of the Sokolow–Lyon criterion for LVH ranges from 4 to 52% and the specificity ranges from 71 to 100%, while similar results were shown by the Cornell index, which, depending on the study, ranges from 2 to 41% while the specificity ranges from 89 to 100% [30]. In existing studies, R_aVL_ was a better predictor of CV events than other ECG criteria, and is also practical for everyday use in clinical practice, including OOM [5,37]. A study by Verdecchia et al., conducted on 2042 patients with AH without CV disease and echocardiographically confirmed LVH who were followed for 7.7 years, found that an increase in R_aVL_ by 0.1 mV indicates a 9% increase in CV risk, while the Cornell index did not show a significant association with CV risk [2]. Additionally, the study showed that patients with AH with a measured RaVL > 0.65 mV had a 1.71% per-year risk of a CV event.

Results from the double-masked, multicentric EWPHE study on 840 patients with AH aged over 60 years (424 placebo and 416 subjects treated with a combination of hydrochlorothiazide and triamterene) showed that AH treatment led to a significant reduction in R_aVL_ compared with placebo after one and four years of follow-up. The reduction in R_aVL_ was associated with a decrease in systolic BP, while changes in BMI did not affect R_aVL_. The importance of this study is also demonstrated by the conclusion that higher R_aVL_ was significantly associated with higher CV and total mortality [38]. Interestingly, this study does not show an association between the Sokolow–Lyon index and mortality. Other studies have monitored changes in ECG criteria for LVH in patients with AH treated with various antihypertensive drugs and have shown a significant reduction in LVH incidence with good blood pressure control [39,40]. According to these studies, ACEi may play the most significant role in reducing the risk of developing LVH and CV risk, but larger studies are needed to confirm these findings and to investigate the effects of established antihypertensive drugs on changes in ECG voltage criteria.

### 4.2. Dipping Profile

Grouped by ABPM results, the largest share of patients with non-dipper and inverse dipper profiles was found in group C. R_aVL_ was significantly higher in non-dippers than in dippers only in this group (0.80 vs. 0.68 mV, *p* = 0.048). Combined with unregulated AH, the absence of a physiological nocturnal BP drop could additionally increase the R_aVL_ in these patients and lead to HMOD. Studies have shown that non-dippers have a double risk of HMOD and CV events compared to dippers [41]. “Apparently healthy” subjects had the lowest R_aVL_ values. However, in 29% of them, a regular nocturnal decrease in blood pressure was absent, which should also be considered. Interestingly, a negative correlation was found between a nocturnal BP drop and R_aVL_ (r = −0.187, *p* < 0.001), but was absent in the linear regression model. On the other hand, nocturnal BP measurement can be imprecise due to the patient’s sleeping position (lying on the cuff), frequent waking up, insomnia, or other factors. In other studies, obesity (which is also the cause of increased R_aVL_) was the most important predictor of having a non-dipper profile and the most common cause of obstructive sleep apnea [42,43,44]. Non-dipper is also more common in male patients with hypercholesterolemia, coronary disease, and hyperuricemia [45]. All of these claims are important for reducing the risk of stroke, which occurs more often in the morning (from 6 am to 12 pm) and can be associated with a non-dipper profile [46]. Because high oxidative stress can accelerate atherosclerosis and lead to HMOD and CV events, recent studies concluded that glutathione peroxidase-loaded nanogels could be the future key players in CV prevention [47].

### 4.3. LDL Cholesterol and Glucose

LDL cholesterol showed a very weak association with R_aVL_ (r = 0.124, *p* = 0.023). Still, this study could not determine the actual influence of LDL cholesterol on R_aVL_ due to its design. Considering these findings, statin therapy (especially atorvastatin) in these patients would have a role in reducing oxidative stress of the myocardium and preventing and reducing LVH, which it achieves through its pleiotropic effects and not through its primary effect on the reduction in LDL cholesterol [48].

Subjects with prediabetes in this study had significantly higher R_aVL_ values than patients without prediabetes (0.57 vs. 0.41 mV; *p* < 0.001). Studies have shown a bidirectional effect of LVH on glucose metabolism, with the presence of LVH being more strongly associated with the occurrence of higher glucose values than higher glucose values being associated with the development of LVH [49,50,51]. A study by Lutale et al. conducted on 271 subjects with type 1 and type 2 diabetes found no association between glucose values and their changes with changes in R_aVL_ or the Cornell index. The prevalence of LVH according to the Sokolow–Lyon criteria was 12.2%, and according to the Cornell criteria was 5.1%, and the values of both criteria were significantly associated with age, blood pressure, and albuminuria, with no difference between subjects with type 1 and type 2 diabetes [52]. No other studies have investigated the association between changes in blood glucose concentration and the R_aVL_ or the Cornell index; however, this study showed a weak but significant positive association between fasting glucose and R_aVL_.

### 4.4. Body Mass Index

In this study, BMI proved to be an independent predictor of an increase in R_aVL_ (β = 0.192, *p* < 0.001), which is in line with the observed observations of Courand et al., who, for BMIs greater than 25 kg/m^2^, presented higher threshold values of R_aVL_ for the assessment of all-cause mortality (>0.8 mV), while for CV mortality, it was the same in all groups according to BMI [5]. Several studies have shown that obesity reduces the sensitivity of ECG criteria that use precordial leads to assess LVH due to the attenuation of detected voltage caused by the greater distance of the electrodes from the heart, which is filled with adipose tissue [53,54,55]. This effect was more pronounced on the Sokolow–Lyon index than on the Cornell index, since R_aVL_, which is not determined by precordial electrodes, is also considered for its calculation. Therefore, Rider et al. propose the Sokolow–Lyon index adjustment by adding 4 mm to the sum for overweight individuals and 8 mm for obese individuals, which significantly increases the index’s sensitivity and specificity in this patient group [56]. This study showed a positive correlation between R_aVL_ and heart rate (r = 0.173, *p* = 0.002), but this association was not significant in the multivariate linear regression model (β = 0.039, *p* = 0.242). Although no studies have linked heart rate to R_aVL_, Inoue et al. found an inverse relationship between heart rate and LVH in males [57].

### 4.5. Strengths and Limitations

Strengths: This study includes subjects in the same age range as those for whom the SCORE 2 model was designed (40–70 years). Only subjects who had not experienced any CV event so far, nor had known CV, diabetes, or other diseases that have a known impact on the results of PWV or R_aVL_ measurements, were included. Among subjects with AH and HU treated in the emergency department, acute HMOD was excluded by standard biochemical and radiological tests, thereby supporting the validity of the R_aVL_ measurements in this group.

Limitations: Because the R_aVL_ results were compared with the SCORE 2 CV risk in high-risk European countries, generalization to the entire European population is not possible. This cross-sectional study cannot determine the actual risk of developing CV disease in patients with higher PWV and R_aVL_ values. Future studies should be prospective, multicenter, and ideally international to draw more precise conclusions regarding the assessment of CV risk using PWV results. Some of the known markers of HMOD were not determined in this study and therefore could not be associated with R_aVL_ values (e.g., Albumin/creatinine) [58,59]. In addition to the unknown exact duration of AH, the duration of use and adherence to antihypertensive and statin therapy are also unknown. A longer duration of untreated AH and poorer adherence are the causes of increased R_aVL_ values, but changes in the parameters used to calculate SCORE 2 CV risk also contribute. AH typically develops years before detection, and there is no tool to assess medication adherence and dosing accuracy, which makes these parameters difficult to determine precisely.

## 5. Conclusions

Subjects with AH and HU had the highest R_aVL_ amplitudes among the studied groups. We demonstrate a significant bidirectional association between increased PWV and elevated R_aVL_, whereas increasing age, MAP, and SCORE 2 CV risk were independent predictors of increased R_aVL_. R_aVL_ also depended on BMI and potassium values. The Sokolow–Lyon index was not identified as an independent predictor of either PWV or R_aVL_, whereas the Cornell index, as expected, only predicted increased R_aVL_. A cut-off value for high CV risk by R_aVL_ > 0.40 mV (across all age groups) was established, with a sensitivity of 58% and specificity of 74%. R_aVL_ may serve as a valuable additional marker for CV risk assessment as well as in family medicine, given its simplicity and rapid determination. However, these findings should be validated in a large-scale, prospective, multicenter international study to more precisely define R_aVL_ cut-off values and evaluate their clinical applicability and benefit.

## Figures and Tables

**Table 1:** Anthropometric, laboratory, and other data of included subjects were divided into groups depending on whether they had arterial hypertension and hypertensive urgency.

Variable		Healthy Subjects (A) N = 105	Hypertensive Subjects Without Hypertensive Urgency (B) N = 134	Hypertensive Subjects with Hypertensive Urgency (C) N = 100	*p*
Anthropometric data and history (±SD)					
Age (years)		54.52 ± 7.74	57.08 ± 7.67	56.68 ± 8.40	0.065
Height (m)		171.70 ± 8.98	173.78 ± 10.25	173.19 ± 10.24	0.321
Mass (kg)		74.05 ± 13.92	85.41 ± 19.50	91.55 ± 18.01	<0.001
BMI (kg/m^2^)		25.07 ± 3.69	28.09 ± 4.79	30.32 ± 4.65	<0.001
Laboratory test results (±SD)					
Hematocrit		0.428 ± 0.031	0.441 ± 0.033	0.438 ± 0.034	0.028
Hemoglobin (g/L)		144.06 ± 17.84	148.56 ± 12.28	147.27 ± 13.98	0.081
Fasting glucose (mmol/L)		5.45 ± 0.61	5.57 ± 0.77	5.90 ± 0.73	0.402
Creatinine (umol/L)		75.61 ± 13.98	77.32 ± 14.72	78.74 ± 17.04	0.538
eGFR (mL/min/1.73 m^2^)		90.48 ± 12.87	90.24 ± 12.91	89.86 ± 15.04	0.936
Potassium (mmol/L)		4.35 ± 0.33	4.32 ± 0.41	4.13 ± 0.43	0.281
Sodium (mmol/L)		140.49 ± 1.89	139.89 ± 1.89	139.37 ± 2.35	0.725
Total cholesterol (mmol/L)		5.84 ± 1.06	5.77 ± 1.18	6.11 ± 1.21	0.968
HDLc (mmol/L)		1.54 ± 0.36	1.42 ± 0.29	1.37 ± 0.29	0.417
LDLc (mmol/L)		3.67 ± 0.98	3.62 ± 1.02	3.91 ± 1.06	0.999
Triglycerides (mmol/L)		1.31 ± 0.75	1.59 ± 0.81	1.79 ± 0.99	0.021
Uric acid (umol/L)		291.89 ± 70.73	334.69 ± 79.75	373.60 ± 98.09	<0.001
SCORE 2 (%)		3.2 [1.8–5.6]	5.8 [3.7–8.7]	10.1 [5.8–13.2]	<0.001 †
Average values (±SD)					
Sokolow–Lyon index (mV)		1.91 ± 0.55	1.93 ± 0.57	2.34 ± 0.66	0.307
Cornell index (mV)		0.96 ± 0.48	1.33 ± 0.54	1.54 ± 0.57	<0.001
R_aVL_ amplitude (mV)		0.22 ± 0.25	0.49 ± 0.27	0.76 ± 0.24	<0.001
PWV (m/s)		7.79 ± 1.10	8.42 ± 0.99	9.50 ± 1.32	<0.001
Number of subjects (%)					
R_aVL_ < 0.4 mV		77 (73.33)	43 (32.09)	7 (7.00)	<0.001 *
R_aVL_ 0.4–0.7 mV		24 (22.86)	60 (44.78)	24 (24.00)	
R_aVL_ > 0.7 mV		4 (3.81)	31 (23.13)	69 (69.00)	
Gender	Male	39 (37.14)	67 (50.00)	58 (58.00)	0.797 *
	Female	66 (62.86)	67 (50.00)	42 (42.00)	

BMI = body mass index; eGFR = estimated glomerular filtration; SD = standard deviation. R_aVL_ = R wave amplitude in aVL electrocardiogram lead; ANOVA, * Chi square test, † = Kruskal–Wallis test.

**Table 2:** Chronically prescribed antihypertensive drugs depending on ABPM groups.

Drugs	Hypertensive Subjects Without Hypertensive Urgency (B) N = 134 (%)	Hypertensive Subjects with Hypertensive Urgency (C) N = 100 (%)	χ^2^ Test	ALL N = 234 (%)
Angiotensin-converting enzyme (ACE) inhibitors	86 (64.18%)	54 (54.00%)	0.116	140 (59.83%)
Angiotensin receptor blockers (ARB)	9 (6.72%)	15 (15.00%)	0.039	24 (10.26%)
Beta blockers	38 (28.36%)	28 (28.00%)	0.952	66 (28.20%)
Calcium channel blockers	75 (55.97%)	58 (58.00%)	0.756	133 (56.84%)
Thiazides and thiazide-like diuretics	42 (31.34%)	35 (35.00%)	0.556	77 (32.90%)
Moxonidine	11 (8.21%)	27 (27.00%)	<0.001	38 (16.24%)
Urapidil	1 (0.74%)	6 (6.00%)	<0.001	7 (2.99%)
Without drugs	21 (15.67%)	25 (25.00%)	0.076	46 (19.66%)
Monotherapy	22 (16.42%)	7 (7.00%)	0.031	29 (12.39%)

**Table 3:** Multivariate linear regression model with R_aVL_ as the dependent variable (R^2^ = 0.494, Adj R^2^ = 0.478, F = 31.558; *p* < 0.001).

Predictor Variable	Standardized		Nonstandardized		t	*p*
	β	St. Err.	β	St. Err.		
Cornell index (%)	0.485	0.066	0.276	0.037	7.358	<0.001
MAP (mmHg)	0.213	0.057	0.006	0.001	3.742	<0.001
PWV (m/s)	0.413	0.074	0.105	0.019	5.557	<0.001
BMI (kg/m^2^)	0.192	0.044	0.013	0.003	4.402	<0.001
SCORE 2 (%)	0.166	0.073	0.012	0.005	2.257	0.025
Potassium (mmol/L)	−0.088	0.041	−0.073	0.035	−2.119	0.035
Age (years)	−0.169	0.083	−0.007	0.003	−2.044	0.042
Gender	−0.079	0.045	−0.053	0.030	−1.752	0.081
eGFR (mL/min/1.73 m^2^)	0.074	0.043	0.002	0.001	1.737	0.083
Intercept			−0.912	0.312	−2.918	0.004

MAP = mean arterial pressure; PWV = pulse wave velocity; BMI = body mass index; eGFR = estimated glomerular filtration rate; St. Err. = standard error.

## Data Availability

The data presented in this study are available on request from the corresponding author.
